# Young leaders doing diabetes education in a school

**DOI:** 10.1186/1758-5996-7-S1-A185

**Published:** 2015-11-11

**Authors:** Matheus Chaluppe de Oliveira, Ronaldo José Pineda Wieselberg, Mark Thomaz Ugliara Barone, Patrícia Vieira de Luca, Viviana Giampaoli

**Affiliations:** 1 Young Leaders In Diabetes (IDF/ADJ), São Paulo, Brazil

## Background

Although type 1 diabetes mellitus (DM) is one of the most common chronic diseases in children, school staff and students know very little about it. Most of what they know are misconceptions, like: “the person who has it cannot eat sugar”, or “it is a disease of old people”. Due to this lack of knowledge, they sometimes refuse to have students with DM, to assist them with their self-care routine, or are unable to help in an emergency. One of the Brazilian Young Leaders in Diabetes (YLD) made, as its conclusion YLD Training project, a diabetes information campaign in his former school.

## Materials and methods

In addition to the speech in 6 classrooms (126 students, grades 8th-12th, ages between 13 and 17 yrs., 44% boys and 56% girls), 4 pamphlets were made and placed on the school dashboards (about: hypoglycemia, DM symptoms, and invitation to the speech). A questionnaire was used before and after the speeches. It contained multiple choice questions about: existence of a family member with DM; appropriate help for someone with DM who is shaking, dizzy, pale and nervous; frequency of sports practice; behaviors that help in preventing type 2 DM; if it is possible to cure DM or not; and if he/she had DM. There was also an open-ended question about DM symptoms. Wilcoxon signed rank test with the continuity correction was used to compare the pre- and post-speech number of right and wrong answers to each question.

## Results

There was only 1 student with T1D. Most of them (63%) reported to have a family member with DM (20% no family member with DM, and 17% did not know). In addition, 49% practice physical activity 3 or more times a week, 29% only once a week, and 22% do not practice. The 4 questions compared pre- and post-speech presented significant difference in terms of higher number of correct answers after the speech, all of them with a p-value < 0.001.

## Discussion

Our objectives were partially achieved, since after the speech most of the students, but not all, answered correctly the questions about: hypoglycemia correction (percentage of right answer pre- and post-speech: 21% and 64%), type 2 DM preventive behaviors (62% and 78%), diabetes symptoms (10% and 71%), and DM possibility of cure (49% and 91%).

## Conclusion

We believe that led by a Young Leader the project may be more effective, because the connection with someone from a closer age group and language may enhance students' interest.

